# State dissociation moderates response to dialectical behavior therapy for posttraumatic stress disorder in women with and without borderline personality disorder

**DOI:** 10.3402/ejpt.v7.30375

**Published:** 2016-07-06

**Authors:** Nikolaus Kleindienst, Kathlen Priebe, Nora Görg, Anne Dyer, Regina Steil, Lisa Lyssenko, Dorina Winter, Christian Schmahl, Martin Bohus

**Affiliations:** 1 Department of Psychosomatic Medicine and Psychotherapy, Central Institute of Mental Health, Medical Faculty Mannheim, Heidelberg University, Germany; 2 Department of Psychology, School of Social Sciences, and Otto-Selz-Institut, University of Mannheim, Mannheim, Germany; 3 Department of Psychology and Sports Sciences, Institute of Psychology, Johann Wolfgang Goethe University, Frankfurt/Main, Germany; 4 Faculty of Health, University of Antwerp, Antwerp, Belgium

**Keywords:** Borderline personality disorder, childhood abuse, dialectical behavior therapy, dissociation, exposure therapy, posttraumatic stress disorder, psychotherapy

## Abstract

**Background:**

Patients with posttraumatic stress disorder (PTSD) are prone to dissociation, which in theory should interfere with successful treatment. However, most empirical studies do not substantiate this assumption.

**Objective:**

The primary objective was to test whether state dissociation predicts the success of an adaptation of dialectical behavior therapy designed for the treatment of patients with PTSD after childhood sexual abuse (CSA) (DBT-PTSD). We further explored whether the operationalization of dissociation as state versus trait dissociation made a difference with respect to prediction of improvement.

**Methods:**

We present a hypothesis-driven *post hoc* analysis of a randomized controlled trial on the efficacy in patients with PTSD after CSA. Regression analyses relating pre–post improvements in the Clinician-Administered PTSD Scale (CAPS) and the Posttraumatic Diagnostic Scale (PDS) to dissociation were applied to the women who participated in the active treatment arm (DBT-PTSD). Multivariate models accounting for major confounders were used to relate improvements in both the CAPS and the PDS to (1) state dissociation as assessed after each treatment session and (2) trait dissociation as assessed at baseline.

**Results:**

State dissociation during psychotherapy sessions predicted improvement after DBT-PTSD: patients with low state dissociation during treatment had a higher chance to show substantial improvement. This relation consistently emerged across subgroups of PTSD patients with and without borderline personality disorder. The operationalization of dissociation as state versus trait dissociation made a difference as improvement was not significantly predicted from trait dissociation.

**Conclusions:**

Dissociation during treatment sessions may reduce success with trauma-focused therapies such as DBT-PTSD. Accordingly, clinical studies aimed at improving ways to address dissociation are needed.

**Highlights of the article:**

- The article investigates whether state dissociation during psychotherapeutic sessions moderates the success of an established treatment for PTSD.
- Patients were much more likely to substantially improve with respect to PTSD symptomatology if state dissociation during psychotherapeutic sessions was low.
- The article suggests that the relation between low dissociation and good response in highly symptomatic patients is stronger than previously thought.
- Future studies investigating the extent to which outcome might be improved when treating dissociation more vigorously than usually are necessary.

The overall efficacy of psychotherapeutic approaches for the treatment of posttraumatic stress disorder (PTSD) is well supported by empirical evidence (for an overview, see Schnyder et al., 2015; Watts et al., 2013). However, a substantial fraction of PTSD patients do not adequately respond to extant treatment programs. This is particularly true for specific subgroups (e.g., for PTSD after childhood sexual abuse (CSA)). According to a recent meta-analysis (Dorrepaal et al., 2014), improvement rates in women with complex PTSD related to childhood abuse are below 50% on an intention-to-treat basis.

One approach to improve current therapies starts with identifying mechanisms related to therapeutic change. While the treatments for PTSD differ in their applied techniques (e.g., imaginal exposure, in vivo exposure, cognitive interventions), they have several strategies and mechanisms in common. These commonalities include modifying cognitions, emotions, the reorganization of memory functions, and the acquisition of information and skills (Schnyder et al., 2015). While current research has focused on the mechanisms related to the change center on information processing and the modification of memory, the exact neuropsychological mechanisms underlying memory change remain controversial. Recently, Lane, Ryan, Nadel, and Greenberg (2015) proposed a theory based on reintegration of new information to activated memory via the process of reconsolidation resulting in permanent changes of memory traces. However, competing models favor memory mechanisms other than reconsolidation. According to Brewin (2015), psychotherapies create new memories that may collaborate or compete with existing memories for the control of behavior. Specifically, Craske (2015) argues that exposure therapy needs to counter the patients’ original expectations, thereby inducing a secondary inhibitory association.

Whatever the exact mechanisms, neurobiological findings suggest that clinically relevant dissociative phenomena are likely to disrupt information processing and memory on various levels. Specifically, in patients with a history of interpersonal trauma state dissociation was associated with hypoactivation of the hippocampus (a region associated with memory reconsolidation), as well as regions associated with emotion processing (e.g., the amygdala, insula, and anterior cingulate cortex (ACC); Krause-Utz et al., 2012). Therefore, dissociation can be linked to hypoactivity in brain regions involved in emotional information processing and memory. On the other hand, current neurobiological models of dissociation suggest increased activity in prefrontal brain areas associated with executive functioning and emotion regulation (Lanius et al., 2010). This evidence regarding the effect of state dissociation on neural correlates of processes central for learning in psychotherapy—namely, processing of emotional information and memory reconsolidation—has been validated by behavioral and psychophysiological studies. One of these studies linked state dissociation to relevant functional deficits using a differential aversive Pavlovian delay conditioning procedure (Ebner-Priemer et al., 2009). This investigation compared emotional aspects of learning (i.e., skin conductance response, valence, and arousal) in healthy controls and patients with borderline personality disorder (BPD) with high and low levels of state dissociation. In contrast with the control groups, BPD patients with clinically relevant levels of state dissociation completely failed to acquire differential conditioning response. These findings are consistent with the results of Mauchnik, Ebner-Priemer, Bohus, and Schmahl (2010), who found that specifically BPD patients with co-occurring PTSD and high state dissociation exhibited deficits in differentiating conditioned danger and the safety signal.

The profound impact of dissociation on acquisition and processing of memories—as found in highly symptomatic clinical populations (e.g., Ebner-Priemer et al., 2009)—contrasts with mixed findings regarding the link between state dissociation and memory functioning in less-symptomatic populations (for an overview, see Giesbrecht, Lynn, Lilienfeld, & Merckelbach, 2008). For example, in students, state dissociation was unrelated to memory encoding (Huntjens, Wessel, Postma, Van Wees-Cieraad, & De Jong, 2015). Therefore, the clinical studies cited above should not be readily extrapolated beyond populations with high levels of dissociation. More generally, it is important to keep in mind that “the concept of dissociation is semantically open and lacks a precise and generally accepted definition” (Giesbrecht et al., 2008, p. 617). Beyond the traditional interpretation of dissociation as cognitive avoidance, dissociation has been associated with pseudo-memories (which might be mediated by heightened levels of interrogative suggestibility), proneness to fantasy, distractibility, cognitive failures, and sleep-related experiences (Giesbrecht et al., 2008).

Although these theoretical and neurobiological findings suggest a negative impact of dissociation on therapeutic response, the current evidence from clinical studies is mixed. Speckens, Ehlers, Hackmann, and Clark (2006) investigated the relation between dissociation and success of imaginal reliving in 44 patients with diverse traumas (mostly accidents). In contrast to their expectation, the authors “did not find any association between response to imaginal reliving and […] dissociation” (p. 337).

Halvorsen, Stenmark, Neuner, and Nordahl (2014) report that dissociative symptoms did “not seem to substantially moderate the treatment of […] Narrative Exposure Therapy […] among severely traumatized asylum seekers and refugees” (p. 26).

The findings of two studies of adult women with PTSD related to interpersonal violence (Resick, Suvak, Johnides, Mitchell, & Iverson, 2012) or childhood abuse (Cloitre, Petkova, Wang, & Lu Lassell, 2012) are more complex. On the one hand, the authors report that the “level of baseline dissociation did not moderate the effect of the treatments on PTSD outcome” (Cloitre et al., 2012, p. 709) and that “overall, pre-treatment levels of dissociation did not impact change in PTSD symptoms” (Resick et al., 2012, p. 727). On the other hand, both studies report that dissociation might differentially affect the treatments under investigation. Cloitre et al. (2012) reported that participants with high dissociation only continued to improve during follow-up when receiving Skills Training in Affective and Interpersonal Regulation followed by Narrative Story Telling (STAIR/NST). Resick et al. (2012) reported a differential impact of dissociation on cognitive processing therapy (CPT) versus CPT-C (the cognitive component of the complete CPT package) in that women with “low pretreatment levels of dissociation responded most efficiently to CPT-C, whereas women with the highest levels of dissociation […] responded better to CPT” (p. 718).

The findings on prolonged exposure (PE) are mixed. In their study of 71 patients who had suffered from diverse traumas, Hagenaars, Van Minnen, and Hoogduin (2010) found that “none of the dissociative phenomena … predicted improvement” after PE (p. 19). However, in 284 female veterans and duty service members treated with PE and present-centered therapy for PTSD, Wolf, Lunney, and Schnurr (2015) found that the subgroup with the dissociative subtype of PTSD reported a significantly “lesser decrease in PTSD severity scores” than patients who did not meet the criteria of the dissociative subtype; however, this effect was small in magnitude. The significance in the study by Wolf et al. (2015) versus Hagenaars et al. (2010) might be related to their substantially larger sample size.

In a recent study, Bae, Kim, and Park (2015) investigated the impact of pretreatment clinical variables on the success of Eye Movement Desensitization and Reprocessing (EMDR) in PTSD patients, most of whom had experienced an accident. Dissociative items of the Clinician-Administered PTSD Scale (CAPS; Blake et al., 1995) were significantly related to the probability of non-response after EMDR; the items of the Dissociative Experience Scale (DES; Bernstein & Putnam, 1986) were not. As further evidenced by this study, patients with a high number of co-occurring disorders were less like to respond to EMDR.

Furthermore, successful treatment of PTSD was found to be negatively related to numbing (Taylor et al., 2001) and alienation (Ehlers et al., 1998) but was found to be promoted via emotional activation and habituation (Jaycox, Foa, & Morral, 1998). The latter study is particularly interesting in light of the findings by Ludäscher et al. (2007), who found that activation of aversive arousal engenders dissociation in BPD patients. Accordingly, emotional activation during exposure therapy might co-activate state dissociation, which, in turn, could inhibit emotional learning.

For some newly developed treatments of PTSD, the potential impact of dissociation on outcome has not been investigated. One of these treatments is DBT-PTSD, a modular, multicomponent treatment program for PTSD that includes both imaginal exposure and elements from DBT (e.g., skills training).

In sum, while some previous studies of psychotherapeutic treatment of PTSD indicated a possibly moderating role of dissociation, they did not consistently support a detrimental effect of dissociation on outcome. One reason for the surprisingly low and inconsistent impact of dissociation might be related to the assessment of dissociation. In the aforementioned studies, dissociation was either assessed at study entry, using scales which assess dissociation as trait, or retrospectively. Hence, the hypothesis that current dissociation, which actually manifests during therapeutic sessions (i.e., state dissociation), interferes with treatment outcome still remains to be tested.

Furthermore, a more systematic investigation of the impact of co-occurring disorders is desirable. As evidenced by Bae et al. (2015), successful treatment of PTSD is made difficult by co-occurring psychiatric disorders. Therefore, effective management of comorbidity merits attention. Especially for patients with co-occurring BPD, dissociation might be a factor related to treatment outcome since BPD patients are prone to dissociation (which is one of the diagnostic criteria of BPD), and empirical evidence supports a negative impact of dissociation on treatment response in patients with a BPD diagnosis (Kleindienst et al., 2011). Hence, dissociation might be especially problematic in PTSD patients with co-occurring BPD.

## Objectives

Our work has several goals. On a confirmatory basis, we test whether an improvement in PTSD symptoms depends on state dissociation during psychotherapeutic sessions of DBT-PTSD. This treatment program was found to be efficacious in patients with PTSD after CSA (Bohus et al., 2013) but has not yet been investigated with respect to response prediction. In theory, the efficacy of the major components of DBT-PTSD (e.g., formal exposure, cognitive restructuring) critically depends on the processing of emotional information and memories that may be downregulated by state dissociation. Since DBT-PTSD specifically addresses the needs of patients with PTSD related to CSA plus co-occurring psychiatric disorders, dissociation might be of even greater relevance in these patients since CSA is associated with both high dissociation and a severe and complex pattern of psychopathology (Wolf et al., 2012). Furthermore, we explore whether the operationalization of dissociation (state vs. trait) makes a difference with respect to predicting improvement, and whether the results also apply to subgroups of patients with and without co-occurring BPD.

## Methods

The present paper presents a *post hoc* analysis of a randomized controlled trial that compared DBT-PTSD and a treatment-as-usual wait-list (TAU–WL) in patients with PTSD after CSA (Bohus et al., 2013). DBT-PTSD, the procedures, and the recruitment process including patient flow are described in the original study (Bohus et al., 2013) and are only briefly summarized.

### Treatment

DBT-PTSD was designed to be applicable for a wide range of patients experiencing PTSD after severe interpersonal violence, including individuals who show a high burden of psychopathology. This is reflected by its modular multicomponent architecture, which allows sufficient flexibility to cover both complex psychopathology and crises within a principle-based structure. The DBT-PTSD study was conducted as a 12-week residential program. These 12 weeks comprise three treatment phases. In Phase I, patients learn to identify their individual strategies for escaping from trauma-related primary emotions (e.g., dissociation, non-suicidal self-injury). Based on these individualized behavioral analyses, they learn to use specific DBT skills to control these behaviors. The focus of Phase II is on trauma-focused cognitive and exposure-based interventions. If patients exhibit strong dissociative features, they are trained to use specific skills in order to balance memory activation and the awareness of being in the present (skills-assisted exposure). In Phase III, patients work on radical acceptance of trauma-related facts and prepare for return to everyday life. All these phases include different modules (e.g., for reducing dissociative symptoms), which are composed according to the individual needs of the participant while following the DBT hierarchy of treatment targets (Linehan, 1993).

Participants received twice-weekly sessions of individual treatment over 12 weeks. The participants further received weekly group treatments including training in skills, self-esteem, mindfulness, and psychoeducation.

### Assessments

Current dissociation was primarily assessed using the Dissociation-Tension-Scale (DSS-4) (Stiglmayr, Schmahl, Bremner, Bohus, & Ebner-Priemer, 2009), which was administered at the end of each psychotherapeutic session, and covered the time during the respective session. The DSS-4 was specifically developed for repeated assessments of state dissociation. It consists of four items covering depersonalization (“I have the impression that my body does not belong to me”), derealization (“I have the impression other people or things around me are unreal”), somatoform dissociation (“I have problems hearing; e.g., I hear sounds from nearby as if they come from far away”) and analgesia (“I have the impression that my body or parts of it are insensitive to pain”). These items were assessed on Likert scales ranging from 0 (“not present”) to 9 (“very strong”). The DSS-4 showed good-to-excellent psychometric properties with respect to inner consistency, reliability, convergent, and differential validity (Stiglmayr et al., 2009).

At baseline, dissociation was further assessed using the short version of the DES (German version FDS-20) (Spitzer, Mestel, Klingelhöfer, Gänsicke, & Freyberger, 2004), which assesses trait dissociation.

Diagnoses of PTSD and co-occurring Axis I disorders were established at baseline using the Structured Clinical Interview for DSM-IV (SCID-I; First, Spitzer, Gibbon, & Williams, 1997). The presence of co-occurring BPD was established using the International Personality Disorder Examination (IPDE; Loranger et al., 1994).

Disorder-specific pathology was assessed using both observer-based and self-rating instruments. The CAPS (Blake et al., 1995) was administered by experienced and specifically trained clinical psychologists. The Posttraumatic Diagnostic Scale (PDS; Griesel, Wessa, & Flor, 2006) was used as primary self-rating. Both scales were assessed with regard to the event that was currently causing the highest distress (“index-trauma”).

Further assessments of psychopathology included the Global Severity Index (GSI) of the Symptom Checklist-90-Revised (SCL-90-R) (Derogatis, 1992), and the Borderline Symptom List (BSL; Bohus et al., 2007).

### Participants

All participants were treated at the CIMH, Mannheim (Germany). Patients were eligible if they fulfilled the following criteria:

1. PTSD related to CSA
2. Co-occurring major depressive disorder (current), and/or substance abuse (current), and/or eating disorder (current), and/or at least four DSM-IV criteria for BPD
3. Female gender
4. Age 17–65
5. None of the following exclusion criteria: BMI<16.5, schizophrenia (lifetime), bipolar-I disorder, substance dependence (current), intellectual disability, medical conditions contradicting the exposure protocol, or life-threatening behavior within the last 4 months
6. Written informed consent

The study was approved by the competent ethics committee and registered with ClinicalTrials.gov (NCT00481000).

As we were interested in the impact of dissociation during psychotherapeutic sessions, state dissociation was only assessed for patients from the active psychotherapeutic group. For external reasons, the DSS-4 was only introduced in March 2008 and therefore was conducted with 28 (out of 36) DBT-PTSD participants. Of these, two participants dropped out before exposure started, and two more participants had to be excluded from the analyses as their ratings of state dissociation were essentially missing (<2 out of 24 scheduled assessments). Accordingly, the analyses are based on the data of 24 participants.

### Statistical analysis

To avoid spurious results, we first plotted the distributions of all data. As no abnormalities (e.g., outliers) were detected, we applied parametric tests such as correlations according to Bravais–Pearson.

The major hypothesis, that patients with higher state dissociation would show less improvement in PTSD-specific symptomatology by the end of treatment, was tested using linear regression analyses. Improvement was operationalized as pre-to-post difference in one of the primary outcomes, that is, the Δ-scores in the CAPS and in the PDS. Given the rather small sample size, we used a robust two-step procedure to address the interdependence of repeatedly measured state dissociation and treatment effects. First, we analyzed individual state dissociation during both sessions with and without formal exposure by using the following intra-individual model (with *i* indicating the patient and *j* indicating the session number): *dissociation*_*ij*_=*intercept*_*i*_+*slope*_i_*session_*j*_+*e*_*ij*_. While *slope*_*i*_ indicates the trend of dissociation over the sessions, *intercept*_*i*_ indicates the individual level of dissociation after controlling for this trend. Controlling for this trend is important as it absorbs the confounding effect of DBT-PTSD on the DSS scores during the treatment phase. In the second step, the dissociation indexes were entered as predictors in the final regression model. We further included baseline severity of PTSD symptomatology as independent variable, because baseline severity is linked to both the level of dissociation and the treatment effect. Accordingly, the potentially confounding effects of baseline severity of PTSD symptomatology were controlled for (for a detailed discussion of how to model Δ-scores, see Barnett, Van der Pols, & Dobson, 2005).

To explore whether the operationalization of dissociation as state dissociation versus trait dissociation impacts the results, we further used a regression model which included trait dissociation (DES scores at baseline) again controlling for baseline severity.

Analyses were based on all patients who had sufficient data to define the variables used in the respective models. Pre–post effect sizes (*d*) were calculated as mean differences standardized with the maximum likelihood estimate of the difference. *P*-values≤0.05 (two-tailed) were considered statistically significant. Analyses were carried out using SAS™ (v.9.4).

## Results

The mean age at study entry in the sample of 24 patients was 37.3±10.5 years (range: 19–52). The patients fulfilled an average of 4.0±1.8 BPD criteria, 16 (67%) met at least four criteria and nine (38%) met full diagnostic criteria for BPD. At study entry, the mean scores were 88.9±15.3 (for the CAPS), 2.26±0.46 (PDS), 2.13±0.74 (BSL), 1.93±0.73 (GSI), and 31.4±18.7 for the DES (range: 2.1–68.3).

On average, patients attended 22.3 individual psychotherapeutic sessions (range: 19–26). The mean DSS-4 scores were 3.06±2.35 during the sessions prior to the start of formal exposure, 3.00±2.34 during exposure sessions, and 2.26±2.38 for the sessions following exposure. The DSS-4 score averaged across all sessions (2.58±2.18) was significantly correlated with the DES scores assessed at study entry (*r*=0.71). The mean improvements from study entry to post-therapy were 34.1±28.5 (*d*=1.20) on the CAPS, 0.67±0.68 (*d*=0.99) on the PDS, 0.67±0.72 (*d*=0.93) on the BSL, 0.61±0.65 (*d*=0.94) on the GSI, and 10.4±14.7 (*d*=0.71) on the DES.

**Fig. 1:**
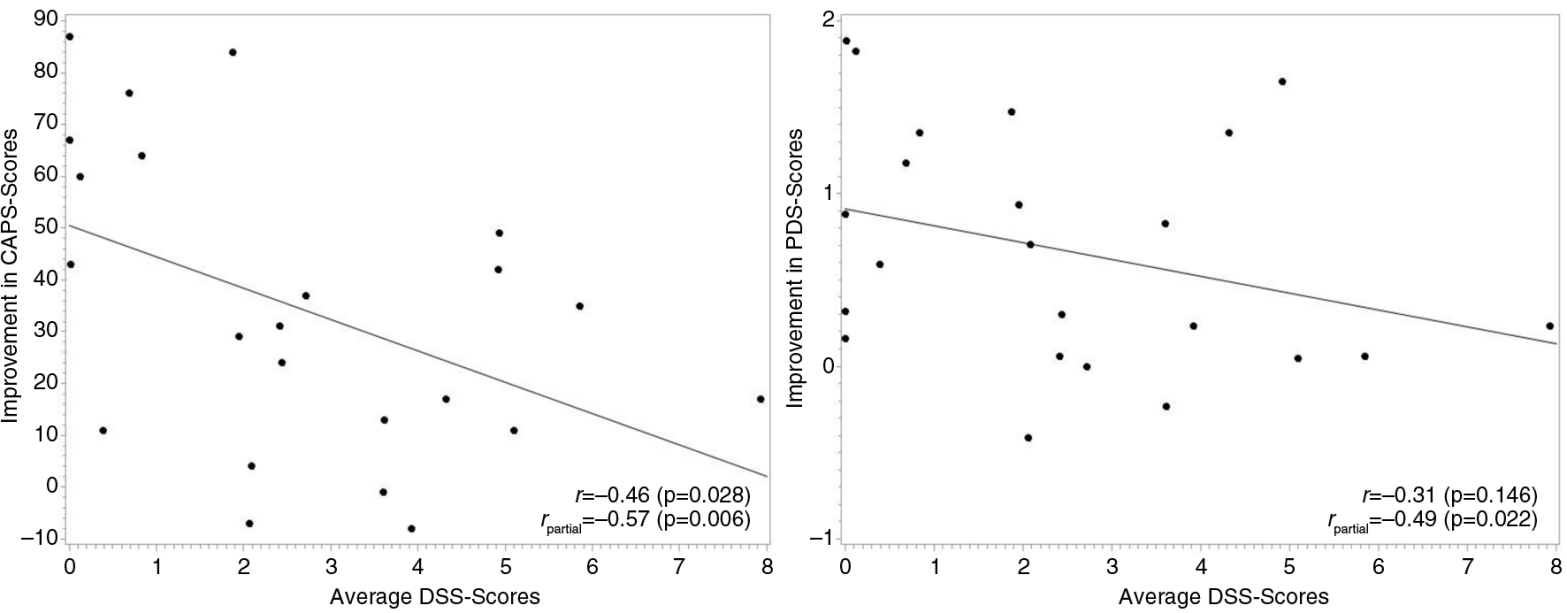
Scatterplots of improvements in the primary outcome variables (ΔCAPS and ΔPDS scores) against the mean scores of state dissociation during all psychotherapeutic sessions. *r* is the raw correlation between the Δ-scores and dissociation; *r*_partial_ is the partial correlation between those two variables while controlling for severity of PTSD symptoms at baseline.

As shown in Fig. 1, the correlation between the average DSS-4 scores and the primary outcome variables tended to be negative (*r*=−0.46, *p=*0.03 for ΔCAPS and *r*=−0.31, *p*=0.15 for ΔPDS scores). Partial correlations controlling for severity of PTSD symptoms at baseline (a major confounder) confirmed the negative relations between average DSS-4 scores and the primary outcome variables (*r*_partial_=−0.57, *p*<0.01 for ΔCAPS and *r*_partial_=−0.49, *p*=0.02 for ΔPDS scores). As further shown in Fig. 1, the negative correlation between dissociation and outcome did not fully offset the treatment effect in patients with moderate or elevated levels of dissociation. While patients with low DSS-4 scores profited most, patients with elevated DSS-4 still improved. However, the differences between patients with low versus elevated DSS-4 scores were clinically meaningful – the estimated improvements in the CAPS were as high as 50 in patients with DSS-4 scores of 0, and dropped to 34 and 18 in patients with DSS-4 scores of 3 and 6, respectively.

These results were confirmed when further controlling for the trend of dissociation over time using the final regression model explained in the Methods section. State dissociation was again negatively related to both the CAPS and the PDS (*p*=0.001 and 0.011, respectively, see Table 1) after controlling for change of state dissociation over time and for baseline severity. This finding establishes an inverse association between state dissociation and outcome, and hereby supports the principal hypothesis of this article.

**Table 1:** Regression of pre- to post-treatment improvements (ΔCAPS and ΔPDS) on the level of state dissociation while controlling for major confounders

Pre–post differences in the CAPS scores regressed on the level of state dissociation during all psychotherapeutic sessions while controlling for (1) the change of state dissociation over time and (2) baseline severity in the CAPS. Overall *F*_3,19_=8.28, *p=*0.001			

	Parameter estimate (mean±standard error)	*t*	*p*

*α*: intercept	−36.108±26.302	−1.37	0.186
*β*_1_: level of state dissociation	−6.873±1.831	−3.75	0.001
*β*_2_: change of state dissociation over time	−136.29±39.20	−3.48	0.003
*β*_3_: baseline severity (CAPS)	1.001±0.293	3.41	0.003
			
Pre–post differences in the PDS scores regressed on the level of state dissociation during all psychotherapeutic sessions while controlling for (1) the change of state dissociation over time and (2) baseline severity in the PDS. Overall *F*_3,19_=4.18, *p*=0.020			

	Parameter estimate (mean±standard error)	*t*	*p*

*α*: intercept	−0.465±0.614	−0.76	0.459
*β*_1_: level of state dissociation	−0.157±0.056	−2.79	0.011
*β*_2_: change of state dissociation over time	−3.295±1.111	−2.97	0.008
*β*_3_: baseline severity (PDS)	0.671±0.292	2.29	0.033

The models further indicate that a decline in both the CAPS and PDS is related to a decline in state dissociation (*p*= 0.003 and 0.008, respectively, see Table 1). This relation might reflect positive effects from getting control over dissociation during the therapeutic process or might simply reflect the intercorrelation of general PTSD psychopathology and dissociation.

The final regression models were also carried out for the subgroups of participants with and without a diagnosis of BPD. These subgroup analyses yielded consistent results – improvements in the CAPS were significantly related to lower levels of state dissociation for both patients with and without a diagnosis of BPD (*β*_1_=−8.57±2.94, *p*=0.033, and *β*_1_=−7.01±3.06, *p*=0.045, respectively). Improvements in the PDS scores were significantly related to lower levels of state dissociation in BPD patients (*β*_1_=−0.05±0.01, *p*=0.009); in patients with no diagnosis of BPD, this relation was in the same direction, but not statistically significant (*β*_1_=−0.01±0.01, *p*=0.122).

**Fig. 2:**
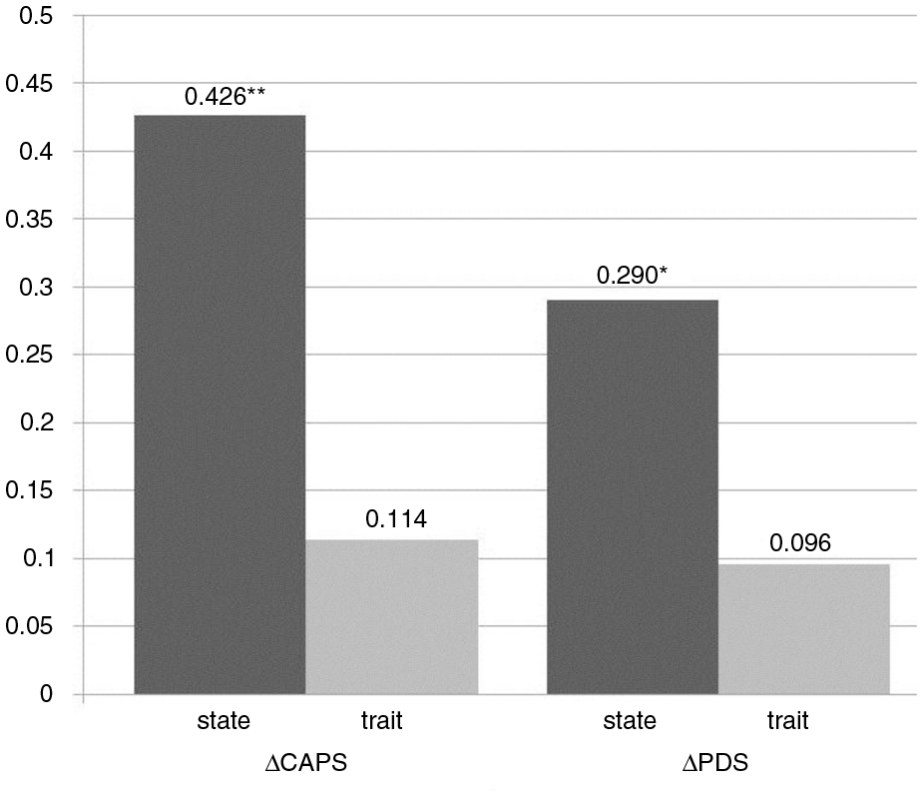
Proportions of variance explained when regressing improvement (in the CAPS and PDS, respectively) on state versus trait dissociation within multiple regression models.

As both trait dissociation and state dissociation were assessed in one study, this provided us with the opportunity to explore whether the operationalization of dissociation as state dissociation versus trait dissociation made a difference with respect to prediction of improvement. Accordingly, we supplemented our primary analyses on state dissociation with a model relying on trait dissociation as predictor while controlling for the potentially confounding effects of baseline severity. Trait dissociation was not a significant predictor for both the ΔCAPS and ΔPDS scores (*β*=−0.488±0.312, *p*=0.135, and *β*=−0.013±0.009, *p*=0.172, respectively). While the variances explained by the mean level of state dissociation are statistically significant and correspond to large effect sizes (proportions of variance explained of 0.426 and 0.290, respectively), the proportions of variance explained by trait dissociation are much smaller and are not significant (see Fig. 2). However, the question of whether the operationalization of dissociation as state dissociation versus trait dissociation has an impact on the results cannot be conclusively answered from our study, as the formal comparison of the respective partial *r* ^2^-values were not statistically significant (*z*=1.34, *p*=0.180, and *z*=0.88, *p*=0.378, respectively).

## Discussion

The results of this study confirm the hypothesis that the level of state dissociation during psychotherapeutic sessions is inversely related to improvements after DBT-PTSD. While improvement was observed across all levels of dissociation, patients were much more likely to substantially improve with respect to PTSD symptomatology if dissociation during treatment was low.

This finding extends previous results in several ways. It confirms and complements the results by Wolf et al. (2015) and Bae et al. (2015); these authors, at least partially, reported an inverse relation between dissociation and improvement after psychotherapeutic treatment of PTSD. However, our study is the first investigation to establish this relation for DBT-PTSD. Furthermore, the effect was substantially larger than in previous studies.

Our results also complement the mixed (mostly negative) findings by Cloitre et al. (2012), Hagenaars et al. (2010), Halvorsen et al. (2014), Resick et al. (2012), and Speckens et al. (2006). Our results indicate that the exact definition of dissociation might affect the results of empirical studies. This is supported by our secondary analyses since the link between dissociation and improvement was specific to state dissociation and did not emerge when trait dissociation was used instead. While this result supports our preferred interpretation of the discrepant findings, our study is not conclusive in this respect as the differences between the models using state versus trait dissociation as predictors were substantial, but not significant.

Our study clearly differs from previous studies in aspects such as the treatment investigated and patient characteristics. While the current stage of knowledge does not allow us to isolate or exclude factors that might explain the differences between our findings and previous research, we do not favor the interpretation that our results are specific to DBT-PTSD. Similar to the treatments that were investigated by other groups, DBT-PTSD includes trauma-focused, exposure-based, and cognitive techniques and incorporates several anti-dissociative features (e.g., specific skills training to improve awareness of being in the present). Similarly, we do not believe that the high percentage of BPD patients in our sample accounts for the major findings of our study. This is evidenced by our subgroup analyses in which state dissociation was inversely related to improvements on the CAPS in both PTSD patients with and without co-occurring BPD. Still, patients from our sample scored higher than patients in most other samples with respect to PTSD-specific symptomatology (mean CAPS score of 88.9) and dissociation (mean DES score of 31.4). Since higher psychopathology scores are typically associated with more variance in these scores, it is conceivable that the relationship between dissociation and improvement after therapy emerges more readily in highly symptomatic samples.

Limitations of our study include the observational, not experimental design, which precludes inferences about a causal influence of dissociation. Ultimately, we aim to improve behavioral therapies such as DBT-PTSD by identifying mechanisms related to change. However, our study takes only an initial step in this direction. Another limitation is our rather small sample size, which requires the use of robust statistical methods and reduces statistical power. However, statistically significant results emerging from small studies such as ours imply quite large effect sizes, which, in turn, have more clinical significance. While *post hoc* power analyses indicated adequate statistical power for the primary outcome (0.94 for the CAPS and 0.77 for the PDS), the limited power related to the small sample size precluded a formal comparison of the BPD versus non-BPD subsamples. With respect to external validity, the findings from our sample cannot be readily generalized to the entire population of PTSD patients. Specific aspects that might prevent a simple generalization include the type of trauma (exclusively CSA), female gender, and high symptom severity. Furthermore, our findings only apply to patients who completed the treatment; the potentially confounding effects of attention deficit hyperactivity disorder (ADHD) and co-occurring dissociative disorders have not been investigated. A further limitation is related to our assessment of dissociation. Since we did not assess a wide range of factors related to dissociative symptoms (e.g., quality of sleep, distractibility), it is conceivable that the described relation between dissociation and poor response might have caused or mediated by subtle deficits in neuropsychological performance that might have limited the patients’ cognitive capacity during the treatment sessions. As described, the term “dissociation” is semantically open thus comprising heterogeneous aspects. Accordingly, future research on the link between treatment response and dissociation should co-assess related phenomena such as sleep, fluid intake, and distractibility. This approach will help to identify targets for intervention and may ultimately improve extant therapies. While these limitations affect the external validity and conceptual precision, we do not believe that they affect our main findings. Our result that dissociation during psychotherapeutic sessions negatively predicts improvement is bolstered by the fact that it is consistent with neurobiological findings supporting the idea that acute dissociation attenuates neural plasticity, which is a prerequisite of emotional learning (Ebner-Priemer et al., 2009; Krause-Utz et al., 2012; Winter et al., 2015).

Our study suggests several lines of future research. First, studies investigating the extent to which outcome might be improved when treating dissociation more vigorously than usually are necessary. Second, additional studies about how to treat dissociation (e.g., on how to improve specific skills training) are clearly warranted given the current stage of knowledge. Third, a more precise characterization of dissociative symptoms and related conditions (e.g., sleep) would be helpful to define targets for therapeutic interventions.
